# Loss of protozoan and metazoan intestinal symbiont biodiversity in wild primates living in unprotected forests

**DOI:** 10.1038/s41598-020-67959-7

**Published:** 2020-07-02

**Authors:** Claudia Barelli, Barbora Pafčo, Mattia Manica, Francesco Rovero, Roberto Rosà, David Modrý, Heidi C. Hauffe

**Affiliations:** 1 Department of Biology, University of Florence, Via Madonna del Piano 6, 50019 Sesto Fiorentino, Italy; 2 Department of Biodiversity and Molecular Ecology, Research and Innovation Centre, Fondazione Edmund Mach, Via E. Mach 1, 38010 S. Michele all’Adige, Italy; 3 Tropical Biodiversity, MUSE-Museo delle Scienze, Corso del Lavoro e della Scienza 3, 38123 Trento, Italy; 4 Department of Pathology and Parasitology, University of Veterinary and Pharmaceutical Sciences, Brno, Czech Republic; 5 Institute of Vertebrate Biology, Czech Academy of Sciences, Brno, Czech Republic; 6 Center Agriculture Food Environment, University of Trento, Via E. Mach 1, 38010 S. Michele all’Adige, Italy; 7 Epilab-JRU, FEM-FBK Joint Research Unit, Province of Trento, Italy; 8 Biology Centre, Institute of Parasitology, Czech Academy of Sciences, České Budějovice, Czech Republic; 9 Department of Botany and Zoology, Faculty of Science, Masaryk University, Brno, Czech Republic

**Keywords:** Biodiversity, Conservation biology, Zoology

## Abstract

In light of the current biodiversity crisis, investigating the human impact on non-human primate gut biology is important to understanding the ecological significance of gut community dynamics across changing habitats and its role in conservation. Using traditional coproscopic parasitological techniques, we compared the gastrointestinal protozoan and metazoan symbiont richness of two primates: the Udzungwa red colobus (*Procolobus gordonorum*) and the yellow baboon (*Papio cynocephalus*). These species live sympatrically in both protected and unprotected forests within the Udzungwa Mountains of Tanzania with distinct ecological adaptations and diets. Our results showed that terrestrial and omnivorous yellow baboons had 2 (95% CI 1.47–2.73) and 3.78 (2.62–5.46) times higher gut symbiont richness (both including and excluding rare protozoans) compared to the arboreal and leaf-eating Udzungwa red colobus in unprotected and protected forest, respectively. We also found a consistent depletion of symbiont richness in red colobus living in the unprotected forest fragment compared to the continuous protected forests [the latter having 1.97 times (95% CI 1.33–2.92) higher richness], but not in yellow baboons. Richness reduction was particularly evident in the Udzungwa red colobus monkeys, confirming the pattern we reported previously for gut bacterial communities. This study demonstrates the impact of human activities even on the microbiodiversity of the intestinal tract of this species. Against the background of rapid global change and habitat degradation, and given the health benefits of intact gut communities, the decrease in natural gut symbionts reported here is worrying. Further study of these communities should form an essential part of the conservation framework.

## Introduction

Human exploitation of natural resources and habitats has caused devastating loss of biodiversity, including both macro-^1–3^ and microorganisms^4–6^. Given their critical role in host health and nutrition, the role of bacterial communities, especially the gut microflora, have been a particular focus of attention in recent years, even in wildlife and within a conservation framework^7–9^. However, other relevant components inhabiting the host gastrointestinal tract, such as protozoans and metazoans [hereafter ‘gut symbionts’, since all symbiotic relationships (from mutualism to commensalism to parasitism^10,11^) are possible], did not receive similar attention. Although traditionally considered harmful to the hosts^12^, they are now recognized essential player (i.e. as selective agents^13,14^ and modulators of host behavior^15,16^) and potentially beneficial to host health (i.e. regulating gut homeostasis by restoring bacterial diversity^17^). Thus, ignoring the conservation of this gut component means neglecting a whole set of biological relationships essential for survival, since host–gut symbiont interactions are among the more prevalent ecological and evolutionary drivers of biological diversity and ecosystem composition^18,19^. Several authors have suggested that such gut symbionts should be recognized as meaningful conservation targets along with their hosts^20,21^.

As a result of habitat degradation and/or contraction, studies have revealed that distribution and population size of most animal hosts decreases. If we assume that environmental changes due to human impact similarly affect gut symbionts, we would expect a decrease in prevalence and richness of gut symbionts as well^22–25^. Thus, comparing gut symbiont communities in threatened animal hosts, such as non-human primates^26^ living in both protected and human-impacted habitats, is of particular relevance for understanding the impact of conservation efforts and whole ecosystem health^27^.

The Udzungwa Mountains of Tanzania (hereafter referred as the Udzungwas) are one of 35 internationally-recognized biodiversity hotspots (https://www.conservation.org)^28^; however, intensive agriculture is currently encroaching on these relatively intact forests^29^, making non-human primates living outside protected areas particularly vulnerable^30,31^. We recently discovered that gut metazoan richness of the endemic Udzungwa red colobus monkey (*Procolobus gordonorum*) varies with altitude, with lower richness at lower altitudes^32^. Because human activities are concentrated at lower altitudes, we suggested that anthropogenic disturbance might play an active role in reducing gut symbiont presence and transmission. Therefore, in the present paper, we investigated gut symbiont richness of two sympatric primate species with contrasting life history traits. The endangered Udzungwa red colobus are relatively small canopy dwellers that feed predominantly on leaves^33^; instead, the larger yellow baboons (*Papio cynocephalus*) of least conservation concern, live primarily on the ground and are generalist feeders, with diets including seeds, ripe fruits and animal prey. They are also frequent raiders of human food (i.e. crops and organic waste in villages^34^). Because gut symbiont transmission are likely to depend on host habits and lifestyle, we compared the richness of gastrointestinal protozoans and metazoans of two cercopithecines with highly different ecological adaptations but living sympatrically in contrasting habitat type, predicting higher gut symbiont richness in terrestrial hosts rather than arboreal ones. Moreover, within host species, we predict gut symbiont richness to be higher in hosts living in protected rather than unprotected forest.

## Results

**Figure 1:**
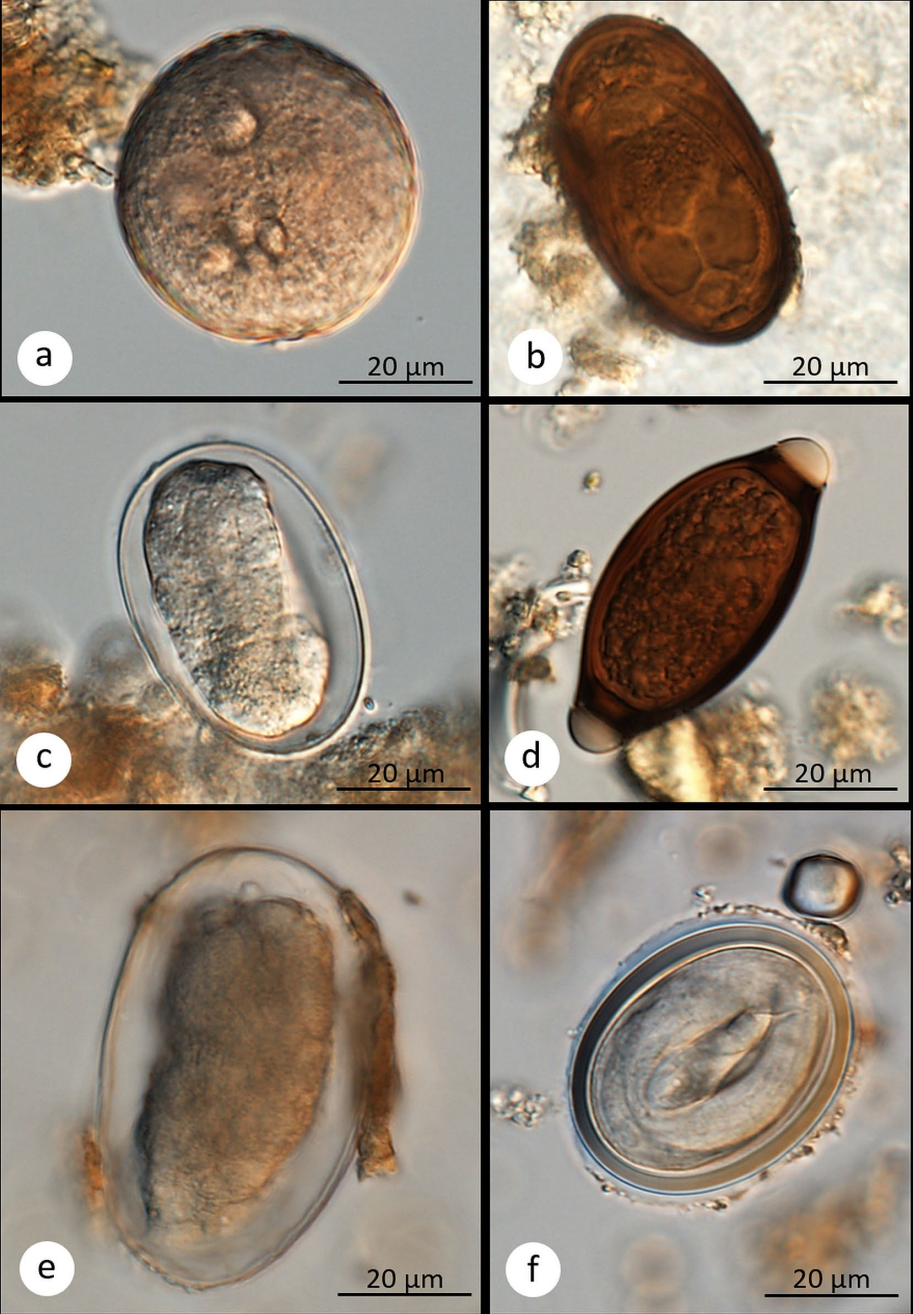
Digital microscopic images of six gut symbionts found in the fecal pellets of yellow baboons and Udzungwa red colobus monkeys from the Udzungwa Mountains of Tanzania: Protozoan: (**a**) cyst of *Balantioides* sp., and eggs of the following metazoans: (**b**) a dicrocoeliid trematode, (**c**) *Strongyloides* sp., (**d**) *Trichuris* sp., (**e**) a strongylid nematode, (**f**) a spirurid nematode.

**Figure 2:**
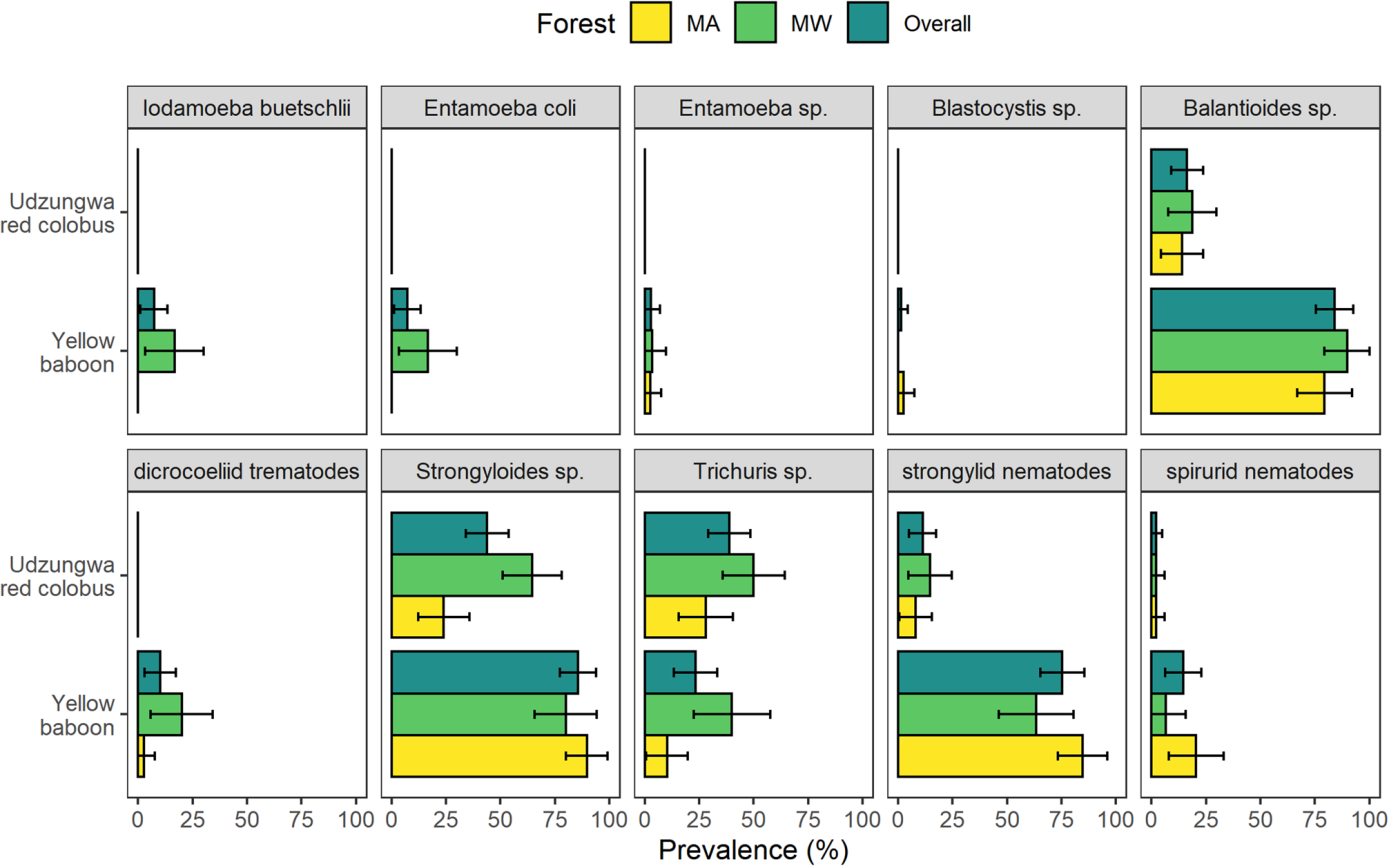
Mean prevalence (expressed in %) of intestinal protozoan and metazoan symbionts in two primate species: Udzungwa red colobus (*Procolobus gordonorum*) and yellow baboon (*Papio cynocephalus*). Colored bars represent the mean symbiont prevalence found from fecal samples of red colobus (N = 69) and yellow baboons (N = 69) collected in two forest types within the Udzungwa Mountains of Tanzania: the unprotected Magombera (MA, colored in yellow) and the protected Mwanihana (MW, light green) forests. Dark green bars indicate the overall mean prevalence for both forest types. Horizontal bars represent the 95% confidence interval.

**Figure 3:**
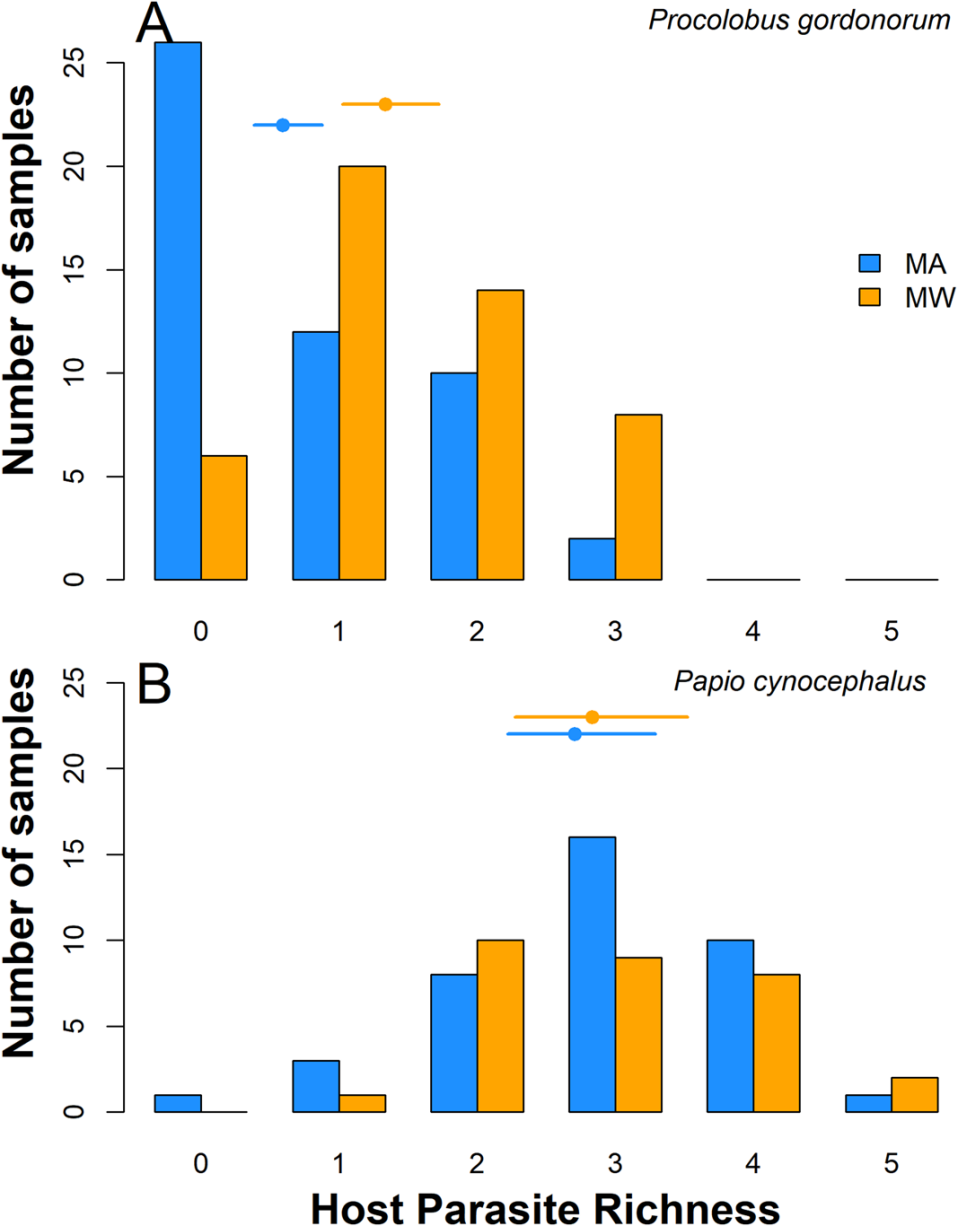
Histogram of the observed gut symbiont richness for two host species, Udzungwa red colobus (*Procolobus gordonorum*) and yellow baboons (*Papio cynocephalus*), in two forests within the Udzungwa Mountains of Tanzania: protected Mwanihana (MW) and unprotected Magombera (MA). Dots represent the expected mean as computed by the Poisson GLM, horizontal bars are the 95% confidence intervals of the mean.

Gut symbionts found in the 167 analyzed fecal samples were categorized into one of 10 taxa (five for protozoans and five for metazoans). Optical microscopy did not always allow for species-level identification of symbiont eggs and cysts; thus, gut symbionts were classified to species (*Entamoeba coli*, *Iodamoeba buetchlii*), genus (*Entamoeba* sp., *Blastocystis* sp., *Balantioides* sp., *Trichuris* sp., *Strongyloides* sp.) or higher taxonomic level (strongylids, spirurids, dicrocoeliid trematodes; Fig. 1). Five of these taxa were observed in both primate species (*Strongyloides* sp., *Trichuris* sp., spirurids, strongylids, and *Balantioides* sp.) with prevalence of each taxa ranging between 2 and 90% (Fig. 2, SI Table S1). The remaining five were present only in baboons (Fig. 2). The majority of samples analyzed (80.2%) contained at least one symbiont. More specifically, all baboon samples but one were infected (98.5%; 68/69), while 67.3% (66/98) of red colobus samples resulted infected (Fig. 3). No metazoan larvae or adults were detected with the gauze-washing method.

### Gut symbiont richness across primate species

In both protected Mwanihana (MW) and unprotected Magombera (MA) forests, yellow baboons had higher mean symbiont richness compared to Udzungwa red colobus, both including and excluding rare protozoans (GLM: Z value: 7.081, df = 136, p value < 0.001; Fig. 3, Table 1; SI Fig. S1, SI Table S2). Based on Binomial GLM analysis, the prevalence of *Strongyloides* sp., strongylid and spirurid eggs, as well as cysts of *Balantioides* sp. were all significantly higher in baboons than in red colobus as reported in Table 2. Instead, *Trichuris* sp. was found in a higher proportion of red colobus samples compared to those of baboons (Binomial GLM: Z value = -1.992, df = 163, p value = 0.0463; Table 2). Shedding intensities revealed that baboons had higher mean eggs/cysts per gram than red colobus (SI Table S3) for both *Strongyloides* sp. (yellow baboons: eggs’ median 434.2, interquantile range 69.1–1,125; red colobus: eggs’ median 63.8, interquantile range 35.9–170; p value = 4.18, df = 100, p value =  < 0.0001) and *Balantioides* sp. (yellow baboons: cysts’ median 762.4, interquantile range 193.7–4,232.9; red colobus: cysts’ median 28.2, interquantile range 22.6–44.4; T value = 6.46, df = 72, p value =  < 0.0001).

**Table 1:** Results of a Poisson Generalized Linear Model (GLM) comparing host symbiont richness (i.e. number of different parasite taxa per fecal sample, including all metazoans and *Balantioides* sp.) between forest type (protected and unprotected), host species (yellow baboon and Udzungwa red colobus) and altitude. Statistically significant values are highlighted in bold.

	Estimate	Se	Z value	Pr( >|z|)
Intercept^a^	− 0.274	0.162	− 1.612	0.0907
Species^b^	1.329	0.188	7.081	< **0.0001**
Forest^c^	0.680	0.201	3.391	**0.0007**
Forest × species	− 0.636	0.245	− 2.592	**0.0095**

Estimates for qualitative variables are provided as mean difference from the reference (Intercept) while for quantitative variables as average unit increases.

Reference levels: ^a^Magombera forest (MA) and Udzungwa red colobus (RC); ^b^YB: yellow baboon; ^c^MW: Mwanihana forest.

**Table 2:** Results of Generalized Linear Models (GLMs) of the prevalence of common protozoan and metazoan symbionts (i.e. number of infected individuals of the whole number of samples examined) in relation to host species, Udzungwa red colobus (*Procolobus gordonorum*) and yellow baboons (*Papio cynocephalus*), inhabiting two contrasting forests (degraded Magombera and intact Mwanihana) and altitude. Statistically significant values are highlighted in bold.

	Predictor	Estimate	Se	Z-value	Pr( >|z|)
*Strongyloides* sp.	Intercept^a^	− 1.153	0.331	− 3.481	0.0005
	Species^b^ (YB)	3.322	0.623	5.331	** < 0.0001**
	Forest^c^ (MW)	1.754	0.448	3.914	** < 0.0001**
	Forest (MW) × species (YB)	− 2.536	0.829	− 3.059	**0.0022**
*Trichuris* sp.	Intercept^a^	− 0.945	0.315	− 2.999	0.0027
	Species^b^	− 1.225	0.615	− 1.992	**0.0463**
	Forest^c^	0.945	0.427	2.211	**0.0271**
	Forest × species	0.819	0.775	1.058	0.2903
Strongylid nematodes	Intercept^a^	− 2.442	0.521	− 4.411	< 0.0001
	Species^b^	4.147	0.685	6.059	** < 0.0001**
	Forest^c^	0.675	0.663	0.598	0.3090
	Forest × species	− 1.833	0.883	− 1.629	0.0380
Spirurid nematodes	Intercept^a^	− 3.892	1.010	− 3.853	0.0001
	Species^b^	2.537	1.085	2.338	**0.0194**
	Forest^c^	0.042	1.429	0.029	0.9600
	Forest × species	− 1.326	1.654	− 0.802	0.4226
*Balantioides* sp.	Intercept^a^	− 1.815	0.408	− 4.454	< 0.0001
	Species^b^	3.170	0.569	5.574	** < 0.0001**
	Forest^c^	0.349	0.550	0.634	0.5260
	Forest × species	0.494	0.911	0.542	0.5880

Estimates for qualitative variables were calculated as the mean difference from the reference (Intercept) while for quantitative variables as mean unit increases.

Reference levels: ^a^Magombera forest (MA) & Udzungwa red colobus (RC); ^b^YB: yellow baboon; ^c^MW: Mwanihana forest.

### Impact of habitat on gut symbiont richness

**Figure 4:**
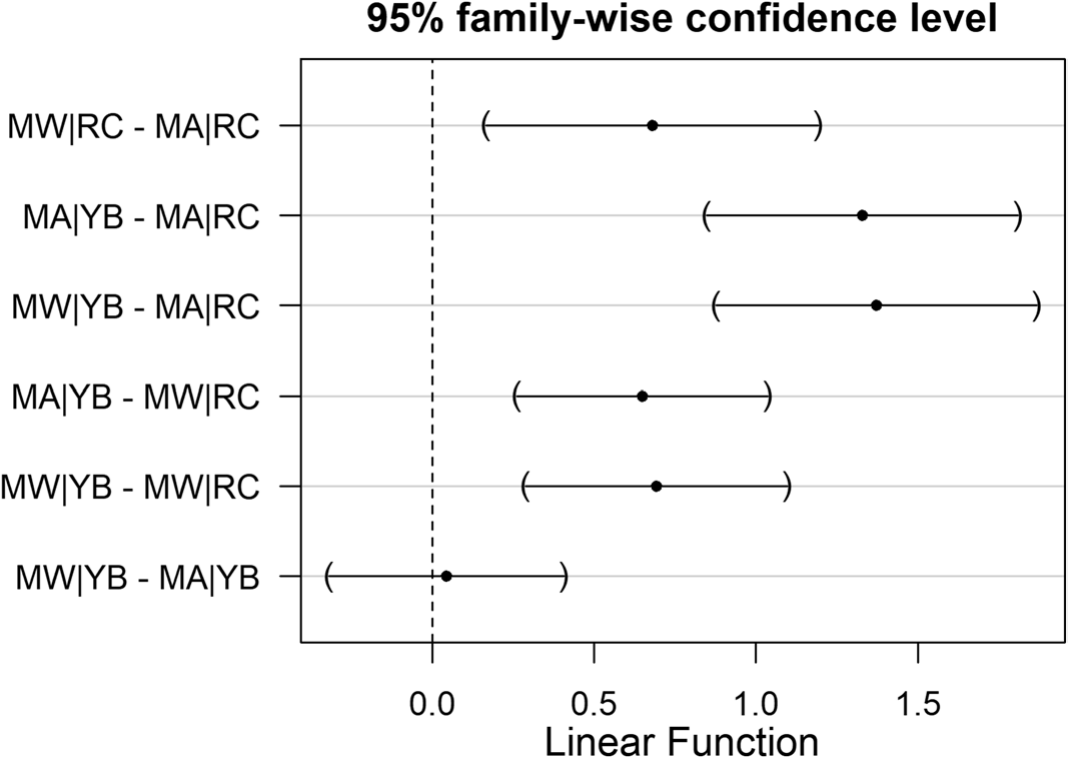
Pairwise comparisons (using Tukey’s HSD for the Poisson GLM) of gut symbiont richness for two host species: Udzungwa red colobus (RC on y-axis) and yellow baboons (YB) living in protected Mwanihana (MW) and unprotected Magombera (MA) forests within the Udzungwa Mountains of Tanzania. On the x-axis the estimated mean values and their 95% confidence intervals (CI) are shown for the estimated difference between group’s parameters tested in each pairwise comparison. On the y-axis the pairwise comparisons are shown. Black dots represent the average difference and horizontal bands represent the 95% CI.

Results from the Poisson GLM (Table 1) suggest that yellow baboons inhabiting MA had 3.78 [95% confidence interval (CI) 2.62–5.46] times more symbionts than Udzungwa red colobus monkeys sharing the same habitat type; while in MW, yellow baboons had about 2 (95% CI 1.47–2.7) times more gut symbionts than red colobus. Moreover, gut symbiont richness of red colobus was on average 1.97 times (95% CI 1.33–2.92) higher in individuals living in MW than in those inhabiting MA. In contrast, the same pattern was not observed in yellow baboons where the difference in symbiont richness between forests was not statistically significant (p value > 0.05; Fig. 4, SI Fig. S2). Gut symbiont distribution and prevalence varied with the taxon investigated. For example, in red colobus, a higher prevalence of *Strongyloides* sp. and *Trichuris* sp. were observed for individuals living in MW compared to MA, but not for the other three taxa (Fig. 2, Table 2; SI Table S1). In baboons, only a higher prevalence of *Trichuris* sp. (Fig. 2, Table 2; SI Table S1) was reported in MW compared to MA. Moreover, from the linear model (SI Table S3), egg shedding intensity of *Strongyloides* sp. resulted to be higher in red colobus in MA (median 85.7, interquantile range 48.6–513.4) than in MW (median 50, interquantile range 31.3–146.4). This trend was not observed in baboons (MA: median 442.6, interquantile range 61.9–1,029.8; MW: median 280.6, interquantile range 86.3–1,357.1).

Finally, modeling showed that overall, altitude influenced neither gut symbiont prevalence nor richness. Only the egg shedding intensity of *Strongyloides* sp. appeared to be influenced by this parameter (SI Table S4).

## Discussion

In this study, we investigated whether habitat degradation impact on protozoan and metazoan richness and prevalence of two wild primate species having different ecological adaptations (terrestrial or arboreal), dietary strategies (omnivorous or folivorous) and living in protected and unprotected forests within an Afrotropical biodiversity hotspot. Through the analysis of non-invasively collected fecal samples, hereafter we compare and discuss the possible reasons why gut symbiont richness and prevalence differ between and within primate hosts. To avoid potential effects of seasonality^35–37^ and altitudinal gradients^32^, fecal samples were collected during a very narrow temporal window from social groups living at similar altitudes; thus, any intraspecific differences should reflect the effects of habitat type. Despite the expected difference between species (with yellow baboons harboring a greater gut symbiont richness compared to the Udzungwa red colobus monkeys), within species, Udzungwa red colobus living in protected forest had a higher level of gut symbiont richness than those living in unprotected forest; this intraspecific difference confirms our previous results for gut bacteria^4,38^.

Although they have been evolving with their hosts for millions of years, until recently protozoan and metazoan gut symbionts have been mainly perceived as pathogens, considered harmful rather than integral to gut homeostasis and host health. However, since gut communities have now been recognized as one of the keystones of the human biome and beneficial against inflammatory mechanisms by manipulating the host immune system^39,40^, they are being considered relevant to the conservation status of their hosts^20^. This paper contributes to the growing body of knowledge revealing micro biodiversity loss in degraded versus intact habitats^4,9,38^, as well as the complex interplay between hosts, symbionts and/or microbiota^21,41–43^.

None of the observed taxa of protozoans and metazoans found in this study were new to non-human primates^44^, having been noted previously for other red colobus [Ugandan red colobus, *Piliocolobus tephrosceles*^45,46^; eastern black-and-white colobus *Colobus guereza* and Angolan black-and-white colobus *C. angolensis*^47^] or other baboons [olive baboons, *Papio anubis*^48^; guinea baboons, *Papio hamadryas papio*^49^; yellow baboons, *Papio cynocephalus*^50^]. We found yellow baboons had a higher gut symbiont richness and prevalence than Udzungwa red colobus. Although highly speculative because accurate data are not available, we believe that group density [i.e. number of individuals per group^51,52^] is unlikely to be responsible for this result, as predicted in other animal hosts^53,54^. In fact, yellow baboons and Udzungwa red colobus populations in this study area are known to have comparable group sizes (approximately 40–60 individuals per social group). Body size has also been purported to explain gut symbiont richness and prevalence^55,56^, and since yellow baboons are larger than red colobus, this morphological difference could partially explain the higher richness in baboons. However, the two primate species also have very different behavioral traits that could account for the differences in gut symbiont diversities. The most likely driving forces here are diet and time spent on the ground, given the differences in specific gut symbionts’ prevalence. The Udzungwa red colobus are predominantly arboreal, occasionally descending to the ground, while yellow baboons spend much of their time travelling on the forest floor and between nearby villages, where they feed on crops and/or on human food waste. These lifestyle differences would explain the higher prevalence of trophically transmitted gut symbionts like spirurid nematodes and dicrocoeliid trematodes in yellow baboons compared to red colobus: both these symbionts are transmitted indirectly through the ingestion of an intermediate host (often arthropods)^44^, and baboons consume more terrestrial arthropods in their diet than the mainly leaf-eating Udzungwa red colobus monkeys^51,52^. Similarly, fecal-orally transmitted protozoans from contaminated soil (e.g., *Entamoeba coli*, *Entamoeba* sp., *Iodamoeba buetchlii*), and associated with domestic animals (*Blastocystis* sp.) were only found in yellow baboons. Another protozoan transmitted through contaminated water (*Balantioides* sp.), as well as two nematode taxa with soil-dwelling free-living stages acquired through the skin (*Strongyloides* sp. and strongylids) were also found significantly more frequently in yellow baboons than red colobus. That these taxa are more likely to be found in terrestrial rather than arboreal hosts has also been observed intraspecifically for chimpanzees *Pan troglodytes*; i.e. individuals that used the ground more frequently for locomotion were more infected^57^.

The only difference in gut symbiont prevalence noted here that is not explained by the arboreal-terrestrial lifestyle was for *Trichuris* sp. Although this taxon is also soil-transmitted, this genus was more prevalent in arboreal Udzungwa red colobus rather than baboons. Another larger study specifically focused on on *Trichuris* sp. in non-human primates was also unable to identify the factors responsible for prevalence in particular primate species, but terrestriality was not one of them^58^. However, *Trichuris* sp. do appear to prefer dense shade which may explain the discrepancy^59^, since red colobus tend to live in deeper shader forest, while baboons spend much of their time on forest edges. The same peculiarity of *Trichuris* sp. may also explain why in both species, this genus was more prevalent in MW than MA.

Studies on the effect of human disturbance on intraspecific variation of gut symbiont richness and prevalence in wild animals still present very mixed results^60^. For example, several studies conducted on primates have found lower gut symbiont richness in intact, undisturbed habitats compared to disturbed and degraded ones (Tana River red colobus, *Procolobus rufomitratus* and mangabey, *Cercocebus galeritus galeritus*^54^; lion-tailed macaque, *Macaca silenus*^61^; redtail guenon, *Cercopithecus ascanius*^62^). However, the same studies were unable to reveal correlations between richness and habitat type on other primate populations (red colobus, *Piliocolobus tephrosceles* and black‐and‐white colobus, *Colobus guereza*^62^). Similarly, no correlation was found between prevalence and habitat type (lion-tailed macaque, *Macaca silenus*^61^; Tana River red colobus, *Procolobus rufomitratu*^54^; red colobus, *Piliocolobus tephrosceles* and black‐and‐white colobus, *Colobus guereza*^62^). On the other hand, similar to our results, in the black howler monkey (*Alouatta pigra*) gut symbiont reduction was clearly associated with habitat degradation^63^, as well as in other mammals such as Australian skinks *Lampropholis guichenoti*^64^ and several sigmodontinae rodent species (*Akodon cursor*, *A. montensis* and *Oligoryzomys*) from Brazil^65^.

The discrepancy in finding correlations between gut symbiont richness and habitat type within each primate species investigated might be explained by the different ecological adaptations of each species. It is likely that host species dependent on forest integrity for its diet like the leaf-eating red colobus are influenced differently from human disturbance. In fact, human presence and intensified activities in the unprotected MA forest may have played a critical role in shaping habitat features over time and consequently reducing gut symbiont presence and potential transmission. For example, logging may have changed the forest structure by increasing forest edges and fragmentation of canopy cover may have promoted increased exposure to wind and sun radiation facilitating drier conditions and reducing survival of free-living stages of gut symbionts, resulting in lower infections of individuals living in such altered habitats. Although speculative, the lower contamination of gut symbionts in degraded habitats, such as the small lowland forest fragment of Magombera, could also potentially be due to the proximity of sugar cane plantations^66^ and cultivated fields which are often treated with pesticides, fertilizers and anti-helminthics.

Although not true for all animal species, the endangered Udzungwa red colobus monkeys have now been shown to suffer biodiversity loss at multiple levels in degraded, unprotected habitats. Not only are there lower population densities of this species in MA^30,67^, but lower taxonomic and functional diversity of gut bacteria were found in individuals living in this unprotected forest^4,38^. Furthermore, the present study confirms that gut symbiont richness is reduced in red colobus inhabiting MA. If such associations are confirmed in other specialist feeders, it is plausible that gut communities could be used as a tool for evaluating individual health and the conservation status of a species, especially in the case of threatened taxa where non-invasive fecal sampling is necessary^68^. However, further studies are required to identify potential interactions between gut components and between these components and host health before these relationships can be used as biomarkers for the wellbeing of individuals and species.

## Methods

### Sampling site and animals

**Figure 5:**
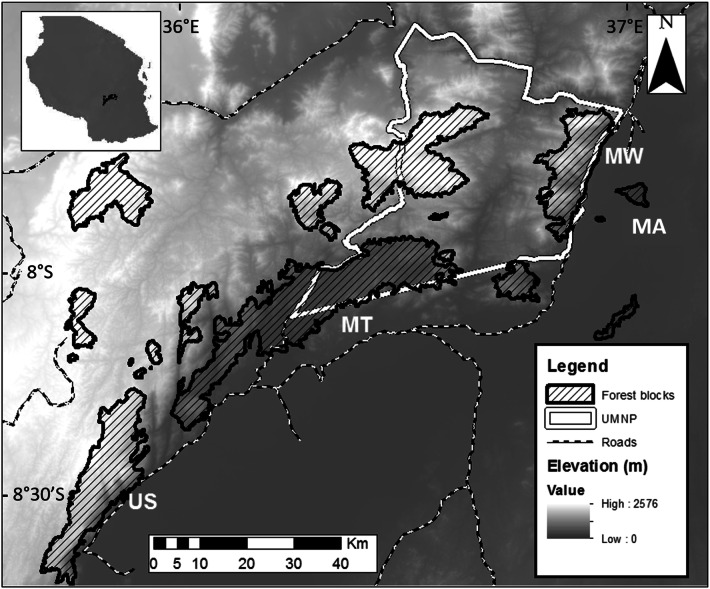
Map of the Udzungwa Mountains in Tanzania showing various forest blocks, including the protected Mwanihana (MW) and unprotected Magombera (MA) considered in this study. The borders of the Udzungwa Mountain National Park (UMNP) are highlighted in white (Modified from Araldi et al. 2014).

The Udzungwas represent the southernmost range of the Eastern Arc Mountains, occupying an area of approximately 19,000 km^2^ (7° 40′ S–8° 40′ S and 35° 10′ E–36° 50′ E; Fig. 5), forming a mosaic of moist forest blocks, interspersed with a matrix of naturally drier habitats, but also croplands and settlements. Rainfall averages 1,500–2,000 mm per year concentrated in two periods: November–December and March–May. Altitude ranges from 270 m a.s.l. (Kilombero valley in the eastern side) to 2,576 m a.s.l. (Mount Luhomero). The protected Mwanihana (MW) and unprotected Magombera (MA) forests were chosen as study sites (Fig. 5; Table 3; see^69^ for details). In the past 50–60 years, these two forests have been gradually separated from each other by at least 6 km of fields and villages^70^. Troops of *Procolobus gordonorum*, the endemic Udzungwa red colobus monkeys, and *Papio cynocephalus*, the yellow baboon live sympatrically in both forests. The Udzungwa red colobus are arboreal and mainly leaf-eating, whereas yellow baboons are terrestrial and omnivorous, feeding regularly on sugar cane as well as human organic waste^51^.

**Table 3:** Characteristics of the protected Mwanihana (MW) and unprotected Magombera (MA) forests in the Udzungwa Mountains of Tanzania.

	MW	MA
Protection level and human presence	National Park (since 1992); several villages along the eastern edge	No formal protection, with a long and complex history of failed attempts of protection; the forest is reducing in size and frequently entered for collecting firewood and hunting
Size (km^2^)	150.6	11.9
Altitude (m)	351–2,263	269–302
Forest type	Lowland, semi-deciduous, sub-montane and montane evergreen forest, including upper montane, bamboo-dominated forest	Ground-water lowland evergreen forest, remaining patches of once continuous forest; surrounded villages and intensive agriculture

Forest size and altitude from Marshall et al. (2010).

### Sample collection

Fecal pellets were collected from 12 red colobus social groups (N = 98 individuals), six (N = 48) from MW, and six (N = 50) from MA. For yellow baboons, samples were collected from five social groups (N = 69), three (N = 30) from MW and two (N = 39) from MA. Most baboon samples were found close to forest edges where baboons regularly crop-raid (especially those from MA). Because red colobus and baboons live in large social groups (~ 40 individuals) and are highly elusive, fecal samples could not be assigned to individuals. However, to avoid any potential re-sampling of the same individual, samples were collected non-invasively during a single defecation event from each group. Social groups sampled during the same day were located at least 2 km apart (more details for sampling procedures in^4,70,71^).

All samples were collected in 2016 during the same 4-week temporal window to avoid potential seasonal effects^36,37^. An aliquot of 2 g of fresh feces was preserved in 10 ml of 10% neutral buffered formalin and stored at ambient temperature (20–25 °C) until transport to the Fondazione E. Mach in Italy where they were kept at 4 °C before definitive shipment of aliquots to the University of Veterinary and Pharmaceutical Sciences in Brno, Czech Republic for parasitological analysis.

### Protozoan and metazoan symbionts examination

Host blood, tapeworm proglottids and adult nematodes that were clearly visible by eye in fecal pellets were noted in the field prior preserving the 167 samples. As recommended for parasitological analyses of non-human primates^44,72^, we used both modified Sheather’s flotation and fecal sedimentation techniques to identify symbionts. First we extracted and weighed sediment from the whole sample, then examined 2 ml of fecal suspension with optical microscopy at 400× magnification (Olympus CX40) for gut symbiont identification. Based on morphological characteristics^44^ eggs and cysts were quantified for each symbiont taxon and the number of eggs or cysts per gram of sediment were calculated according to the following formula: n = N/m, where n = number of eggs/weight of sediment (g), N = number of eggs in examined amount of sediment and m = weight of examined sediment (g). Lastly, using what of the fecal pellet after extraction of sediment, adult and larval nematodes were collected using the ‘gauze-washing’ method^73^ and observed via stereo microscopy at 8–40× magnification (OLYMPUS SZ51).

### Data analyses

Three standard indices of gut symbiont infection were estimated and used as dependent variables in the subsequent regression models:

1. gut symbiont prevalence (i.e. number of infected samples out of the total number of samples examined);
2. gut symbiont richness (i.e. number of different symbiont taxa per sample);
3. gut symbiont egg or cyst shedding intensity (i.e. number of eggs or cysts per gram of fecal sediment for *Strongyloides* sp. and *Balantioides* sp. only).

To assess if the prevalence of each symbiont varied with host species, forest type or the interaction of these two factors, a Generalized Linear Model (GLM) with Binomial distribution and logit link was carried out. Similarly, the association of symbiont richness with primate species, forest type or their interaction was studied using a GLM with Poisson distribution and log link. Because some protozoans (e.g. amoebas and *Blastocystis*) are difficult to detect with optical microscopy^74,75^, the models including symbiont richness were run twice: once with all protozoan taxa, and a second time excluding rare taxa, i.e. where rare is defined as being present in fewer than 10 samples (*Iodamoeba buetschlii*, *Entamoeba coli*, *Entamoeba* spp., *Blastocystis* sp.). Since results from a previous study on the same population of red colobus monkeys revealed that altitude may affect gut symbiont richness in this species^32^, the sampling design was limited to social groups of both species at low altitude (less than 450 m a.s.l.). The altitudinal range in the present study was much narrower (216–441 m) than in the previous investigation (216–1535 m^32^). Therefore, we consider inclusion of altitude in the models by computing the Akaike Information Criteria (AIC) values for each model with or without altitude and decided not to include it (results in Supplementary Information). Gut symbiont diversity measures such as prevalence and richness may depend on infection shedding^76^. Thus, in infected fecal samples, a linear model (LM) was used to assess if the log-transformed egg or cyst shedding intensity for each individual host was associated with primate species or forest type (and their interaction), or altitude. Altitude was standardized (subtracted by its mean and divided by its standard deviation, so to have mean 0 and standard deviation 1). Assessment of the appropriateness of statistical assumptions for each model was carried out by computing overdispersion for GLM and graphically inspecting model residuals^77^. All analyses were carried out in R environment (version 3.5.1; R Core Team 2018).

### Ethics statement

The authors confirm they did not interact with or disrupt any of the primate species surveyed in any way. Fecal sample collection was non-invasive, without direct contact or interaction with the animals. Highly trained fieldworkers strictly adhered to the ‘Code of Best Practices for Field Primatology’ published by the International Primatological Society (IPS) as well as the ‘Principles for the Ethical Treatment of Primates’ of the American Society of Primatologists (ASP). Data collection complied with legal requirements and laws governing wildlife research in Tanzania. Research permits (2016-267-ER-2009-49) were obtained through the Tanzania Commission for Science and Technology (COSTECH), Tanzania Wildlife Research Institute (TAWIRI) and Tanzania National Parks (TANAPA).

## Supplementary information

Supplementary Figure S1

Supplementary Figure S2

Supplementary information

## Data Availability

Data are available in the Figshare repository at https://figshare.com/s/3fe0e6a0bada2a508ad3.
