# Supplementary figures and images for: Loss of protozoan and metazoan intestinal symbiont biodiversity in wild primates living in unprotected forests
Source: Sci Rep. 2020 Jul 2;10:10917. doi: 10.1038/s41598-020-67959-7 (PMC7331812; doi:10.1038/s41598-020-67959-7)

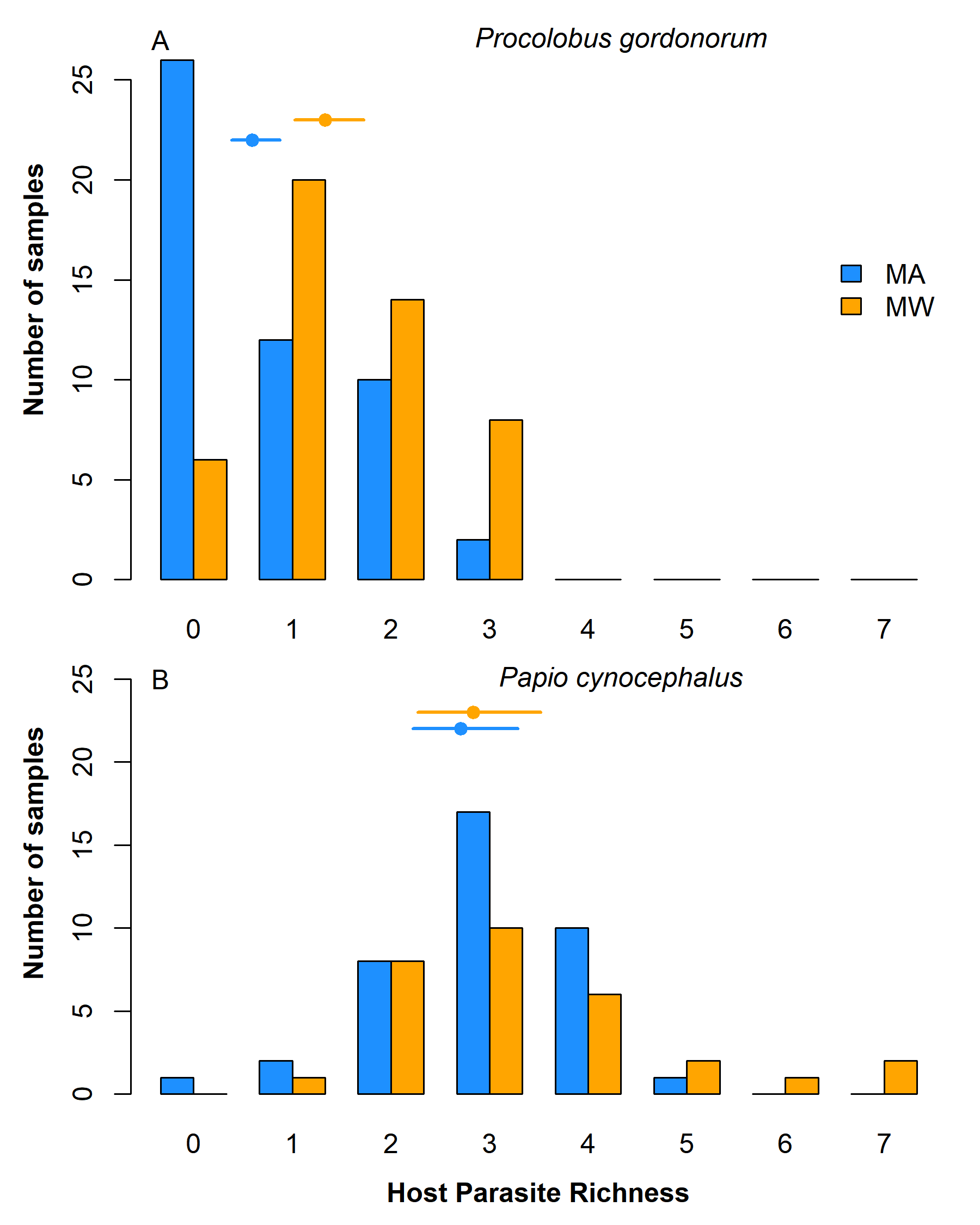

Supplement: Supplementary file 1 — Supplementary Figure S1 [file 41598_2020_67959_MOESM1_ESM.tiff]

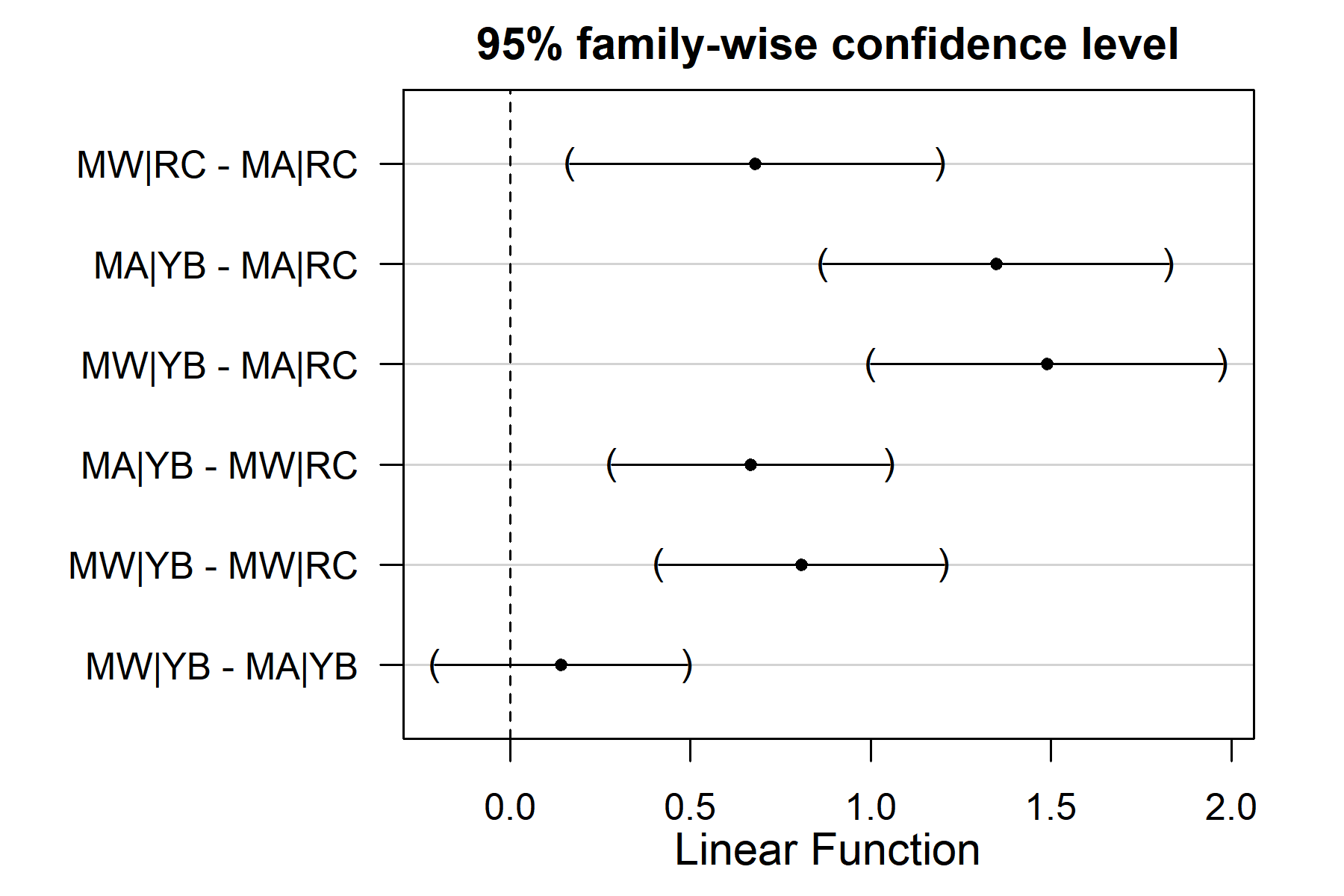

Supplement: Supplementary file 2 — Supplementary Figure S2 [file 41598_2020_67959_MOESM2_ESM.tiff]
