# Supplementary material for: Loss of protozoan and metazoan intestinal symbiont biodiversity in wild primates living in unprotected forests
Source: Sci Rep. 2020 Jul 2;10:10917. doi: 10.1038/s41598-020-67959-7 (PMC7331812; doi:10.1038/s41598-020-67959-7)
Supplement: Supplementary file 3 — Supplementary information [file 41598_2020_67959_MOESM3_ESM.pdf]

Loss of protozoan and metazoan intestinal symbiont biodiversity in wild primates living in unprotected forests

SUPPLEMENTARY INFORMATION

Claudia Barelli[1,2,*], Barbora Pafčo[3,4,°], Mattia Manica[1], Francesco Rovero[2,5], Roberto Rosà[1,6,7], David Modrý[3,8,9] & Heidi C. Hauffe[1,°]

[1] Department of Biodiversity and Molecular Ecology, Research and Innovation Centre, Fondazione Edmund Mach, Via E. Mach 1, 38010 S. Michele all'Adige, Italy

[2] Tropical Biodiversity, MUSE – Museo delle Scienze, Corso del Lavoro e della Scienza 3, 38123 Trento, Italy

[3] Department of Pathology and Parasitology, University of Veterinary and Pharmaceutical Sciences, Brno, Czech Republic

[4] Institute of Vertebrate Biology, Czech Academy of Sciences, Brno, Czech Republic

[5] Department of Biology, University of Florence, Via Madonna del Piano 6, 50019 Sesto Fiorentino, Italy

[6] Center Agriculture Food Environment, University of Trento, Via E. Mach 1, 38010 S. Michele all'Adige, Italy

[7] Epilab-JRU, FEM-FBK Joint Research Unit, Province of Trento, Italy

[8] Biology Centre, Institute of Parasitology, Czech Academy of Sciences, České Budějovice, Czech Republic

[9] Department of Botany and Zoology, Faculty of Science, Masaryk University, Brno, Czech Republic

*Corresponding author; °Authors have contributed equally

Table S1. Overall prevalence (expressed in %) of gastrointestinal protozoan and metazoan gut symbionts in yellow baboon (*Papio cynocephalus*) and Udzungwa red colobus (*Procolobus gordonorum*) from the protected Mwanihana (MW) and unprotected Magombera (MA) forests within the Udzungwa Mountains of Tanzania.

**Symbionts**	**Yellow baboon**			**Udzungwa red colobus**		
	MW	MA	Overall	MW	MA	Overall
Protozoans						
Iodamoeba buetschlii	16.7	0	7.2	-	-	-
Entamoeba coli	16.7	0	7.2	-	-	-
Entamoeba spp.	3.3	2.6	2.9	-	-	-
Blastocystis sp.	0	2.6	1.4	-	-	-
Balantioides sp.	90	79.5	84.1	18.8	14	16.3
Metazoans						
dicrocoeliid trematodes	20	2.6	10.1	-	-	-
Strongyloides sp.	80	89.7	85.5	64.6	24	43.9
Trichuris sp.	40	10.3	23.2	50	28	38.8
strongylid nematodes	63.3	84.6	75.4	14.6	8	11.2
spirurid nematodes	6.7	20.5	14.5	2.1	2	2

Table S2. Results of Poisson GLM of gut symbiont richness for all protozoan and metazoan taxa (i.e., number of different symbiont taxa found per sample) between protected Mwanihana (MW) and unprotected Magombera (MA) forests, primate species (Udzungwa red colobus, *Procolobus gordonorum* and yellow baboon, *Papio cynocephalus*) and altitude. Estimates for qualitative variables are provided as average difference from the reference (Intercept) while for quantitative variables, as average unit increases.

	**Estimate**	**Se**	**Z-value**	**Pr(>|z|)**
Intercept [a]	-0.274	0.162	-1.612	0.0907
Species [b]	1.347	0.187	7.192	***<0.0001***
Forest [c]	0.680	0.201	3.391	***0.0007***
Forest * Species	-0.539	0.243	-2.220	***0.0264***

[a] Reference level: Magombera forest (MA) & Udzungwa red colobus (RC); [b] YB: yellow baboon; [c] MW: Mwanihana forest.

Table S3. Results of Linear Models (LM) comparing log-transformed *Strongyloides* sp. and *Balantioides* sp. eggs/cysts' shedding intensity (i.e., number of eggs/cysts per gram of collected feces) among two primate species, Udzungwa red colobus (*Procolobus gordonorum*) and yellow baboons (*Papio cynocephalus*), inhabiting two contrasting forests (degraded Magombera and intact Mwanihana) within the Udzungwa Mountains of Tanzania. Estimates for qualitative variables are provided as average difference from the reference (Intercept) while for quantitative variables as average unit increases.

		**Estimate**	**Se**	**t-value**	**Pr(>|t|)**
Strongyloides sp.	Intercept [a]	5.121	0.474	10.804	<0.0001
	Species [b]	0.914	0.534	1.710	0.0904
	Forest [c]	-1.767	0.686	-2.577	***0.0115***
	Altitude	0.617	0.232	2.648	***0.0095***
	Forest * Species	1.879	0.803	2.341	***0.0213***
Balantioides sp.	Intercept [a]	3.519	0.689	5.111	***<0.0001***
	Species [b]	3.562	0.762	4.672	***<0.0001***
	Forest [c]	-0.245	0.918	-0.267	0.7906
	Altitude	-0.532	1.036	-0.514	0.6091
	Forest * Species	3.519	0.689	5.111	***<0.0001***

[a] Reference level: Magombera forest (MA) & Udzungwa red colobus (RC); [b] YB: yellow baboon; [c] MW: Mwanihana forest.

Table S4. Summary Table of AIC values for each model with and without altitude. In italics the selected model. When the difference in AIC values was less than 2 the model with fewer parameter was selected.

**Models**	**With altitude**	**Without altitude**
Poisson GLM Richness	489.2	*487.2*
Binomial GLM *Strongyloides* sp.	183.3	*181.3*
Binomial GLM *Trichuris* sp.	199.1	*200*
Binomial GLM strongylid nematodes	150.6	*184.7*
Binomial GLM *Balantioides* sp.	155.5	*153.9*
Binomial GLM spirurid nematodes	80	*81.8*
Egg's LM *Strongyloides* sp.	*391.7*	396.1
Egg's LM *Balantioides* sp.	303.6	*304.7*

List of supplementary figure legends.

Figure S1. Histogram of gut symbiont richness (including all protozoans and metazoans) for two host species, (A) Udzungwa red colobus (*Procolobus gordonorum*) and (B) yellow baboon (*Papio cynocephalus*) from two forests within the Udzungwa Mountains of Tanzania: Magombera (MA, unprotected: blue bars) and Mwanihana (MW, protected: orange bars). Dots represent the expected mean as computed by the Poisson GLM, horizontal bars are the 95% confidence intervals of the mean.

Figure S2. Pairwise comparisons (using Tukey's HSD for the Poisson GLM) for gut symbiont richness (including all protozoans and metazoans) of two host species: Udzungwa red colobus (RC) and yellow baboons (YB) living in two forests: Mwanihana (MW, protected) and Magombera (MA, unprotected) within the Udzungwa Mountains of Tanzania.
